# Sarcoidosis and neuroendocrine tumours: case report and literature review

**DOI:** 10.1002/rcr2.784

**Published:** 2021-05-27

**Authors:** Muhanad Taha, Lobelia Samavati

**Affiliations:** ^1^ Department of Pulmonary Critical Care & Sleep Division Wayne State University Detroit MI USA

**Keywords:** Carcinoid tumour, sarcoidosis

## Abstract

Rare cases of co‐existing sarcoidosis and carcinoid tumour have been previously reported in the literature. Both diseases may have vague and overlapping clinical presentations that can lead to delayed or missed diagnosis. To avoid this diagnostic pitfall, we discuss and compare the clinical presentations of all reported cases in the literature including our case. We also provide hypothesis to explain the relationship between the two diseases.

## Graphical abstract

In the current report, we describe a rare case diagnosed histologically with sarcoidosis and neuroendocrine tumour. We also describe 23 similar cases previously reported in the literature. We discuss the clinical presentations of the cases and possible hypothesis explaining the association between the two diseases. Although co‐existence of these two diseases is extremely rare, understanding the common clinical presentations and the relationship between the two diseases is important as the diagnosis can easily be missed.

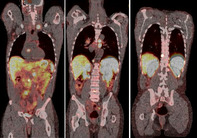

## Introduction

Sarcoidosis is a multisystem granulomatous disease of unclear aetiology [1]. Sarcoidosis typically affects the lung but almost any organ can be involved [1]. The association between sarcoidosis and different types of malignancies has been previously described [2, 3, 4, 5, 6, 7, 8, 9, 10]. Carcinoid or neuroendocrine tumours (NETs) are rare tumours and association with sarcoidosis has been reported in the literature [11, 12, 13, 14, 15, 16, 17, 18, 19, 20, 21, 22, 23, 24, 25]. NETs can arise from any organ but most commonly from lung and small intestine [26]. Only a small percentage of patients with the carcinoid syndrome present with the classical features characterized by the release of vasoactive substances causing bronchospasm, skin flushing, and diarrhoea [26].

In the current report, we describe a rare case diagnosed histologically with sarcoidosis and NET. We also describe 23 similar cases previously reported in the literature. We discuss the clinical presentations of the cases and possible hypothesis explaining the association between the two diseases. Although co‐existence of these two diseases is extremely rare, understanding the common clinical presentations and the relationship between the two diseases is important as the diagnosis can easily be missed.

## Case Report

**Figure 1:**
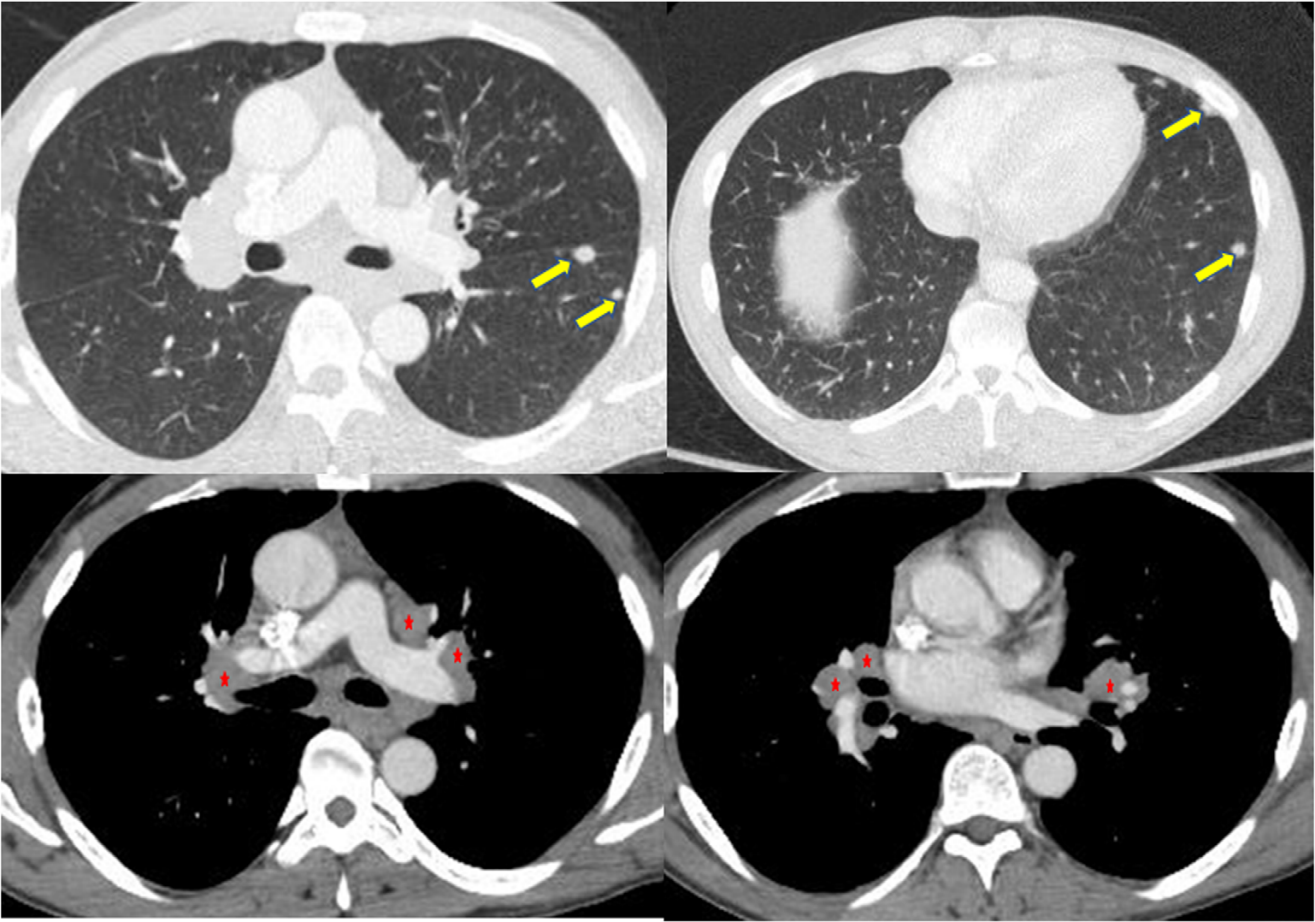
Computed tomography scan of the chest with contrast shows scattered pulmonary nodules (arrows) and hilar and mediastinal lymphadenopathy (stars).

**Figure 2:**
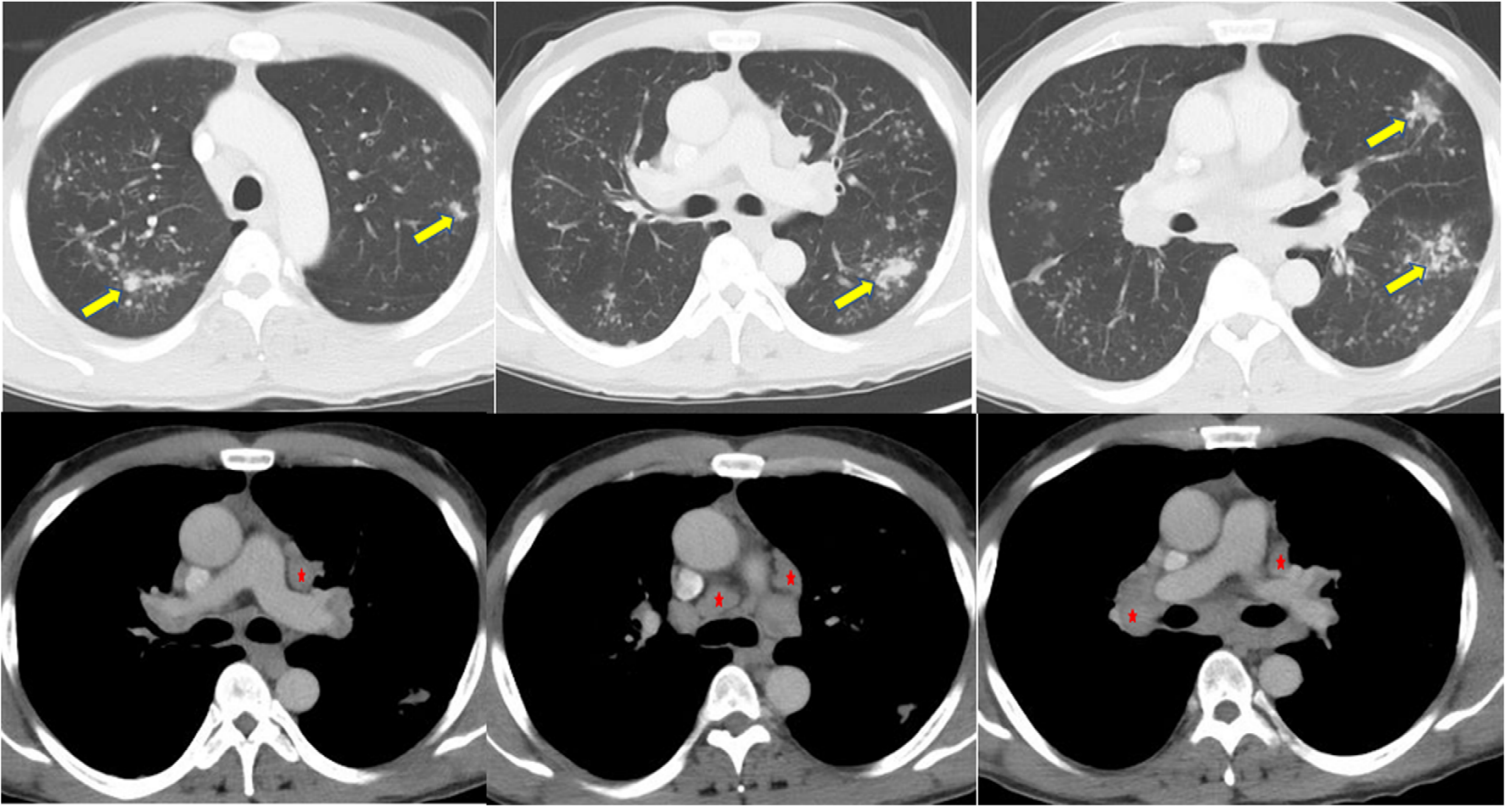
Computed tomography (CT) scan of the chest with contrast, five years after the first CT (Fig. 1), shows scattered pulmonary nodules (arrows) and hilar and mediastinal lymphadenopathy (stars).

**Figure 3:**
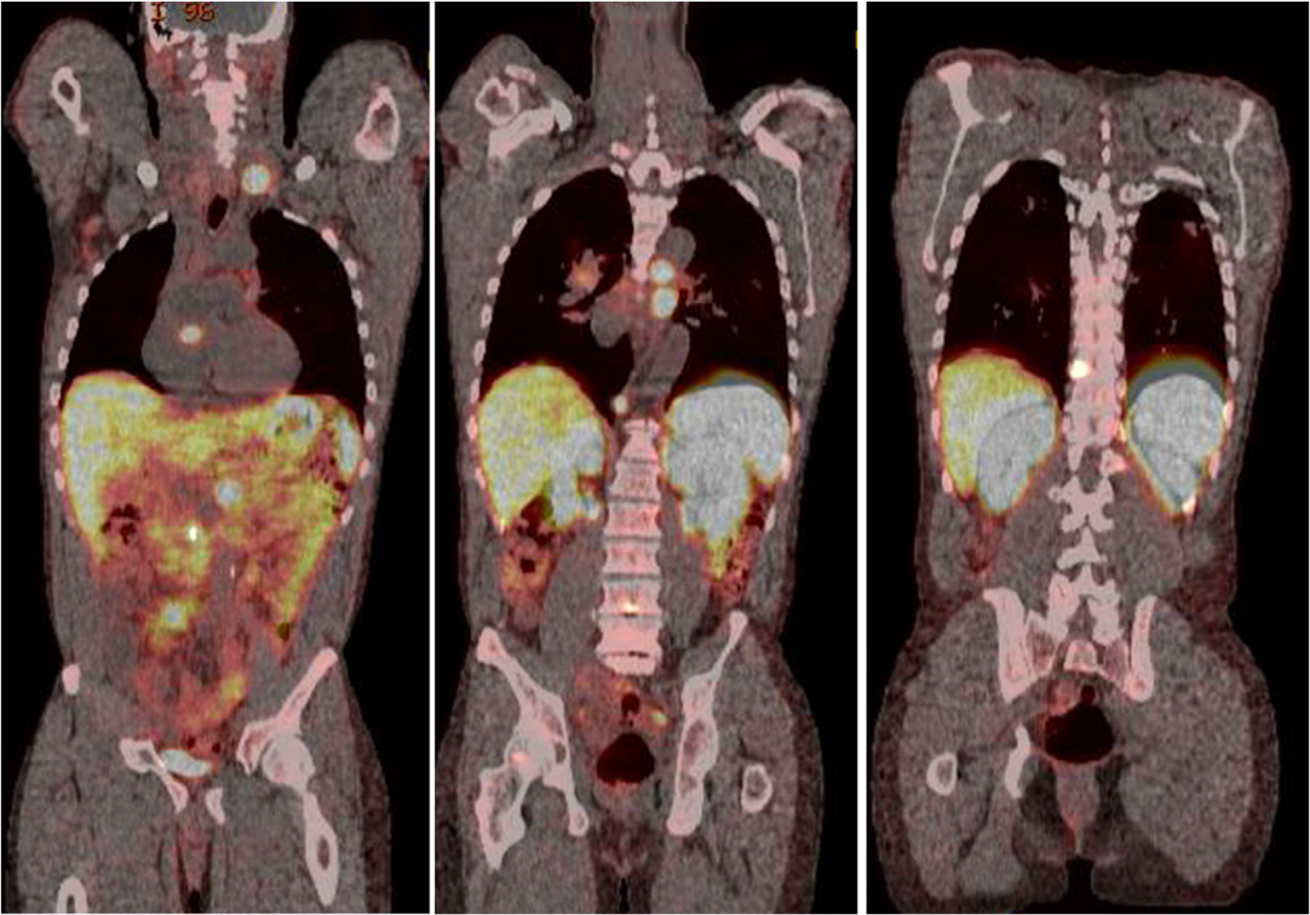
Gallium‐68 Dotatate positron emission tomography (PET)/computed tomography (CT) scan demonstrated metabolically active adenopathy involving the supraclavicular, mediastinum, upper abdomen, and retroperitoneal locations, as well as metabolically active left liver lobe, right small bowel mass, central calcified mesenteric mass, left and right lung nodules, and several lytic metastases in bone.

**Figure 4:**
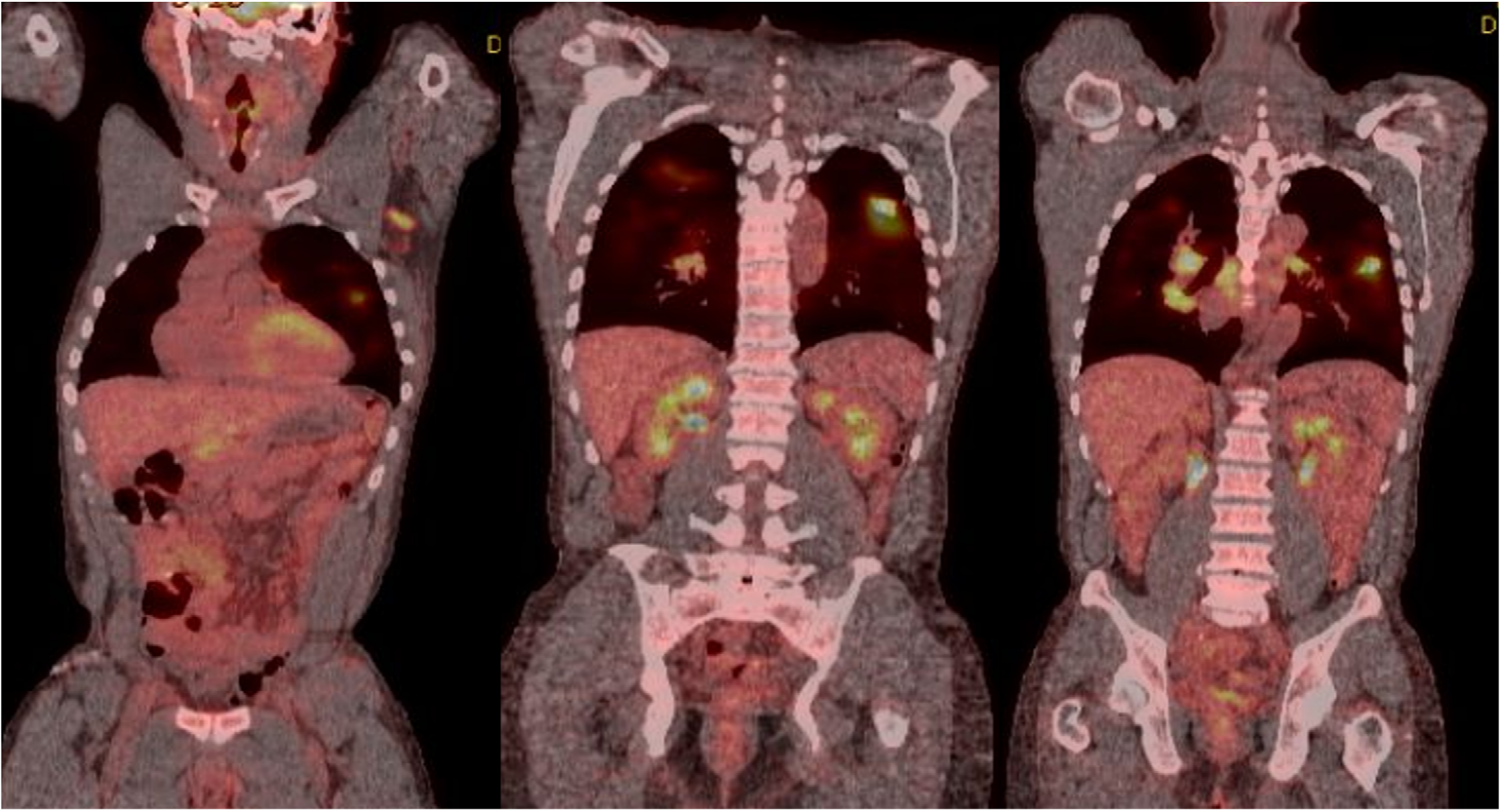
F‐18 fluorodeoxyglucose (FDG) positron emission tomography (PET) demonstrated metabolically active supraclavicular, axillary, mediastinum, and hilar lymph nodes. There was also hypermetabolic activity in the left lung nodule and mesenteric mass in the abdomen.

A 46‐year‐old man presented with abdominal pain and was found to have intestinal intussusception from a mass in the distal ileum. The mass was suspicious for NETs because the patient had chronic diarrhoea and elevated urine level of 5‐hydroxyindoleacetic acid (5‐HIAA) (90.3 mg/24 h, reference range: 0.0–14.9 mg/24 h). Computed tomography (CT) of the chest (Fig. 1) was done as part of the staging workup and demonstrated bilateral subcentimetre nodules with bilateral hilar adenopathy. His abdominal pain resolved with conservative therapy. Further metastatic workup for NETs was planned after discharge; however, the patient was lost to follow‐up. Five years later, the patient presented with a one‐month history of fatigue, cough, and shortness of breath. He also had 40‐pound weight loss over six months. He had persistent chronic diarrhoea and intermittent abdominal pain. He smoked marijuana daily and had a five pack‐year history of smoking. Repeated CT of the chest (Fig. 2) showed left supraclavicular lymphadenopathy, and once again demonstrated bilateral subcentimetre lung nodules and bilateral hilar adenopathy. Biopsy of the hilar lymph nodes revealed non‐caseating granulomas. QuantiFERON‐TB Gold assay was negative. Culture and Gram stain, as well as fungal and mycobacterial culture of bronchoalveolar lavage and tissue specimen were negative. Pulmonary function test (PFT) revealed forced expiratory volume in 1 sec (FEV_1_) of 2.10 L (57% predicted), forced vital capacity (FVC) 3.92 L (86% of predicted), FEV_1_/FVC 54%, residual volume (RV) 2.38 L (104% predicted), total lung capacity (TLC) 6.61 L(90% predicted), and diffusing capacity of lung for carbon monoxide (DLCO) 16.6 mL/mmHg/min (59% predicted), consistent with a moderate obstruction ventilatory disease with no bronchodilator response. Diagnosis of sarcoidosis was made based on clinical, radiological, and histopathological findings, and after other aetiologies for granulomatous diseases had been ruled out. His bilateral subcentimetre pulmonary nodules were attributed to sarcoidosis. There was no evidence of extrapulmonary organ involvement of sarcoidosis. The patient was started on oral corticosteroids. He was also diagnosed with chronic obstructive pulmonary disease (COPD) given his smoking history and the PFT findings, for which he was started on inhaled bronchodilator therapy. Subsequently, the patient was referred to our sarcoidosis clinic. He continued to have poor appetite, fatigue, wheezing, diarrhoea, and shortness of breath. As weight loss and supraclavicular lymphadenopathy are not typical manifestations of sarcoidosis, surgical lymph node removal of supraclavicular lymph node was undertaken and confirmed the presence of an NET that was positive for chromogranin A and synaptophysin by immunohistochemistry. Semiquantitative proliferative rate (Ki‐67) was <5%. Metastatic workup with gallium‐68 Dotatate positron emission tomography (PET)/CT scan (Fig. 3) showed metabolically active left liver lobe, right small bowel mass, central calcified mesenteric mass, and left and right lung nodule. Also, metabolically active adenopathy involving the supraclavicular, mediastinum, upper abdomen, and retroperitoneal locations, as well as several lytic metastases in bone were observed. This indicated that the small intestine is likely the primary site of NETs with metastases to liver, mesentery, lung, and bone. F‐18 fluorodeoxyglucose (FDG) PET (Fig. 4) was done to differentiate between sarcoidosis and NETs and demonstrated metabolically active supraclavicular, axillary, mediastinum, and hilar lymph nodes. There was also hypermetabolic activity in the left and right lung nodules. The mesenteric mass was positive but other abdominal sites previously demonstrated in the Dotatate PET were negative. Subsequently, the patient was referred to oncology and was started on somatostatin analogue therapy for metastatic NETs. In addition, for his persistent cough and shortness of breath, the patient was also started on methotrexate for sarcoidosis. Six months later, the patient started gaining weight and his symptoms of diarrhoea, abdominal pain, and wheezing have improved as well as his cough and shortness of breath.

## Discussion

We found 24 histologically confirmed cases of sarcoidosis and NETs reported in the literature including our case (Table 1). Sarcoidosis and NETs were mostly diagnosed concurrently (cases 1–3, 9, 11–16, 18, 20–22, and 24) or one to four years apart (cases 4, 5, 7, 8, and 19). In only two cases, sarcoidosis was diagnosed 30 years (case 23) and 40 years (case 6) before the diagnosis of NETs. Patients' age ranged from 24 to 75 years. NETs commonly present with gastrointestinal (GI) and lung involvement; in contrast, sarcoidosis commonly presents with lung involvement. Patients with lung carcinoid presented with respiratory symptoms or were found to have incidental lung nodules on imaging or endobronchial lesions during bronchoscopy. Patients with GI NETs reported carcinoid symptoms (such as flushing, diarrhoea, or bronchospasm) or were found to have incidental abnormalities on imaging or endoscopy. In all case reports, sarcoidosis involved the hilar and mediastinal lymph nodes. In 16 out of the 24 cases, there was no extrapulmonary involvement. The other eight cases (4, 5, 7, 8, 10, 15, 22, and 23) had extrapulmonary sarcoidosis with the skin being involved in all these cases. Three cases (4, 5, and 7) had Lofgren syndrome.

**Table 1:** Cases of sarcoidosis and neuroendocrine tumours reported in literature.

Case	Study	Age/sex	Disease chronology	Carcinoid origin	Metastasis	Pulmonary sarcoidosis	Extrapulmonary sarcoidosis
1	Levy, 1997 [11]	46/M	Concurrent	Lung (nodule)	None	Hilar/mediastinal lymphadenopathy	None
2	Levy, 1997 [11]	37/M	Concurrent	Lung (endobronchial lesion)	None	Hilar/mediastinal lymphadenopathy	None
3	Levy, 1997 [11]	66/F	Concurrent	Lung (nodule)	None	Hilar/mediastinal lymphadenopathy	None
4	Levy, 1997 [11]	59/F	Sarcoid precedes carcinoid by three years	GI	Liver	Hilar/mediastinal lymphadenopathy	Erythema nodosum (Lofgren)
5	Levy, 1997 [11]	65/F	Carcinoid precedes sarcoidosis by two years	GI	Liver	Hilar/mediastinal lymphadenopathy	Erythema nodosum (Lofgren)
6	Levy, 1997 [11]	53/M	Sarcoidosis precedes carcinoid by 40 years	GI	Liver	Hilar/mediastinal lymphadenopathy	None
7	Levy, 1997 [11]	31/F	Sarcoidosis precedes carcinoid by one year	GI	Liver	Hilar/mediastinal lymphadenopathy	Erythema nodosum (Lofgren)
8	Weltfriend, 1989 [12]	74/F	Carcinoid precedes sarcoidosis by four years	GI	None	Hilar/mediastinal lymphadenopathy	Skin
9	Thaler, 2000 [13]	Unknown	Concurrent	GI	None	Hilar/mediastinal lymphadenopathy	None
10	Macleod, 2017 [14]	71/F	Sarcoidosis precedes carcinoid	Lung (nodule)	None	Hilar/mediastinal lymphadenopathy	Skin
11	Macleod, 2017 [14]	31/M	Concurrent	Lung (endobronchial lesion)	None	Hilar/mediastinal lymphadenopathy	None
12	Macleod, 2017 [14]	64/F	Concurrent	Lung (endobronchial lesion)	None	Hilar/mediastinal lymphadenopathy	None
13	Avram, 2006 [15]	24/M	Concurrent	Lung (endobronchial lesion)	None	Hilar/mediastinal lymphadenopathy	None
14	La Rosa, 2007 [16]	39/M	Concurrent	Kidney	None	Hilar/mediastinal lymphadenopathy	None
15	Bae, 2010 [17]	38/F	Concurrent	Lung (endobronchial lesion)	None	Hilar/mediastinal lymphadenopathy	Skin and eye
16	Watanabe, 2011 [18]	57/M	Concurrent	Mediastinal mass	None	Hilar/mediastinal lymphadenopathy	None
17	Rafiei, 2015 [19]	42/M	Carcinoid precedes sarcoidosis	GI	Liver, diaphragm, and peritoneum	Hilar/mediastinal lymphadenopathy	None
18	Lera Álvarez, 2012 [20]	43/M	Concurrent	Lung (endobronchial lesion)	None	Hilar/mediastinal lymphadenopathy	None
19	Bhowmik, 2013 [21]	75/F	Carcinoid precedes sarcoidosis by one year	Lung	None	Lung	None
20	Kanapilly, 2014 [22]	50/F	Concurrent	Lung (nodule/mass)	None	Hilar/mediastinal lymphadenopathy	None
21	Mungo, 2017 [23]	52/M	Concurrent	GI	None	Hilar/mediastinal lymphadenopathy	None
22	Lolli, 2017 [24]	34/F	Concurrent	GI	None	Hilar/mediastinal lymphadenopathy	Skin and eye
23	AlSamman, 2018 [25]	62/F	Sarcoidosis precedes carcinoid by 30 years	Lung (nodule/mass)	None	Hilar/mediastinal lymphadenopathy	Skin
24	Taha, 2020 (present case)	46/M	Concurrent	GI	Liver and lung	Hilar/mediastinal lymphadenopathy	None

GI, gastrointestinal.

Gallium‐68 Dotatate PET is the standard technique for evaluating primary and metastatic NETs [15]. Gallium‐68 Dotatate is a radiotracer that binds to somatostatin receptor which is highly expressed in NETs [15]. In patients with co‐existing NETs and sarcoidosis, it is difficult to distinguish between the two entities using gallium‐68 Dotatate PET as sarcoidosis may also express somatostatin receptors and demonstrate high activity on gallium‐68 Dotatate PET [19, 27]. Therefore, granulomatous lesions may be falsely diagnosed with metastatic NETs [19, 27]. A recent case report showed that using F‐18 FDG PET, in addition to gallium‐68 Dotatate PET, may differentiate granulomatous lesions from NETs [19]. F‐18 FDG is a glucose analogue used in PET scan to detect tissue uptake of glucose; therefore, it detects hypermetabolic state of cells due to either cancers or inflammation, including sarcoidosis granulomatous lesions [28]. In our case, F‐18 FDG PET had failed to differentiate between NETs and granulomatous lesions, as numerous regions were expressed by both types of radiotracers. Therefore, further studies are needed to identify the sensitivity and specificity of using F‐18 FDG PET to differentiate sarcoidosis from NETs.

Our literature review showed that metastatic workup of GI NETs may show hilar‐mediastinal lymphadenopathy due to granulomatous reaction that can be mistaken for metastatic NETs. It is important to know that GI NETs can metastasize to supraclavicular or axillary lymph nodes but unlikely to hilar‐mediastinal lymph nodes. In contrast, lung NETs often metastasize to hilar‐mediastinal lymph node; however, granulomatous reaction should also be considered in differential diagnosis.

In patients with sarcoidosis, supraclavicular or axillary lymphadenopathy and GI masses are uncommon manifestations and warrants further investigation to rule out malignancy. However, careful evaluation and biopsy are needed to avoid misdiagnosis.

The proximity in time between the diagnosis of sarcoidosis and NETs raises the question about the relationship between the malignancy and sarcoidosis. One hypothesis is that tumour antigens induce local sarcoidosis‐like reaction in genetically predisposed patients [8, 9, 10]. Sarcoid‐like granulomatous reaction in primary tumour or its draining lymph nodes is documented in the literature [8, 9, 10, 28]. This hypothesis may explain cases (1–3, 11–13, 16, and 18–20) in whom the lung carcinoid tumours (lung nodule, endobronchial lesion, or mediastinal mass) presented with sarcoidosis in locally drained lymph nodes (hilar‐mediastinal lymph nodes). Further support for this hypothesis is that regional sarcoidosis lymphadenopathy has been reported to resolve after surgical removal of carcinoid tumour without any sarcoidosis treatment.

This hypothesis cannot explain why cases (4–9, 14, 17, 21, and 22) with GI NETs have sarcoidosis in hilar‐mediastinal lymph nodes but not in mesenteric lymph nodes where the GI lymphatics drain. This raises an assumption that lymph nodes are not genetically equally predisposed to form sarcoidosis in response to foreign antigens.

The second hypothesis is that tumour antigens can induce systemic sarcoidosis in genetically predisposed patients [8, 9, 10]. Systemic sarcoidosis following diagnosis of malignancy has also been reported in the literature [8, 9, 10]. This may explain why cases (4, 5, 7, 8, 10, 15, 22, and 23) with carcinoid tumours of GI or lung had systemic sarcoidosis with extrapulmonary involvement. Both hypotheses may explain why most sarcoidosis and NETs are diagnosed concurrently or few years apart.

The third hypothesis is that sarcoidosis increases the risk of malignancy because of chronic inflammation in the affected organs and/or immune dysregulation. Studies provide inconsistent results regarding the incidence of malignancy in sarcoidosis patients from no risk to significantly elevated risk [2, 3, 4, 5, 6, 7]. This hypothesis may explain two cases (6 and 23) in whom NETs was diagnosed 30 or 40 years after sarcoidosis. However, in those two cases, the possibility exists that NETs were present at the time of the initial diagnosis of sarcoidosis.

In conclusion, hilar‐mediastinal lymph nodes spread is not typical for NETs and sarcoidosis should be in the differential diagnosis. Also, supraclavicular lymphadenopathy and GI masses are not typical manifestations of sarcoidosis and warrant further investigations to rule out malignancy. We believe that there is a real association between the two entities. We hypothesize that carcinoid tumours induce local and or systemic sarcoidosis. However, further research is needed to understand the relationship between sarcoidosis and NETs and other cancers.

### Disclosure Statement

Appropriate written informed consent was obtained for publication of this case report and accompanying images.

### Author Contribution Statement

Muhanad Taha: Study conception and design, acquisition of data, analysis and interpretation of data, drafting of manuscript. Lobelia Samavati: Study conception and design, critical revision.

## References

[1] Thomas KW , and Hunninghake GW . 2003. Sarcoidosis. JAMA. 289(24):3300–3303. 12824213 10.1001/jama.289.24.3300

[2] Brincker H , and Wilbek E . 1974. The incidence of malignant tumours in patients with respiratory sarcoidosis. Br. J. Cancer 29:247–251. 4830144 10.1038/bjc.1974.64 PMC2009094

[3] Askling J , Grunewald J , Eklund A , et al. 1999. Increased risk for cancer following sarcoidosis. Am. J. Respir. Crit. Care Med. 160(5 Pt 1):1668–1672. 10556138 10.1164/ajrccm.160.5.9904045

[4] Ji J , Shu X , Li X , et al. 2009. Cancer risk in hospitalized sarcoidosis patients: a follow‐up study in Sweden. Ann. Oncol. 20(6):1121–1126. 19211624 10.1093/annonc/mdn767

[5] Ungprasert P , Srivali N , Wijarnpreecha K , et al. 2014. Is the incidence of malignancy increased in patients with sarcoidosis? A systematic review and meta‐analysis. Respirology 19(7):993–998. 25138430 10.1111/resp.12369

[6] Ungprasert P , Crowson CS , and Matteson EL . 2017. Risk of malignancy among patients with sarcoidosis: a population‐based study. Arthritis Care Res. (Hoboken) 69(1):46–50. 27214645 10.1002/acr.22941 PMC5121095

[7] Pavic M , Debourdeau P , Vacelet V , et al. 2008. Sarcoidosis and sarcoid reactions in cancer. Rev. Med. Interne 29(1):39–45. 18054124 10.1016/j.revmed.2007.09.041

[8] Tana C , Giamberardino MA , Di Gioacchino M , et al. 2013. Immunopathogenesis of sarcoidosis and risk of malignancy: a lost truth? Int. J. Immunopathol. Pharmacol. 26(2):305–313. 23755746 10.1177/039463201302600204

[9] Cohen PR , and Kurzrock R . 2007. Sarcoidosis and malignancy. Clin. Dermatol. 25(3):326–333. 17560310 10.1016/j.clindermatol.2007.03.010

[10] Brincker H . 1995. Sarcoidosis and malignancy. Chest 108(5):1472–1474. 7587468 10.1378/chest.108.5.1472

[11] Levy NT , Rubin J , DeRemee RA , et al. 1997. Carcinoid tumors and sarcoidosis – does a link exist? Mayo Clin. Proc. 72(2):112–116. 9033542 10.4065/72.2.112

[12] Weltfriend S , Harth Y , and Katz I . 1989. Subcutaneous sarcoidosis in a patient with malignant carcinoid tumor of the colon. J. Am. Acad. Dermatol. 20(3):507–508. 2465328 10.1016/s0190-9622(89)80092-1

[13] Thaler W , Winkler M , Fichtel G , et al. 2000. Carcinoid tumor of the rectum associated with sarcoidosis: case report with review of the literature. Onkologie 23:362–364.

[14] Macleod JL , Goizueta A , Stanchina M , et al. 2017. Ambiguity of the interplay between carcinoid and sarcoidosis. Am. J. Respir. Crit. Care Med. 195:A3389.

[15] Avram AM , Mackie GC , Schneider BJ , et al. 2006. Differentiation between carcinoid and sarcoid with F‐18 FDG PET and In‐111 pentetreotide. Clin. Nucl. Med. 31(4):197–200. 16550010 10.1097/01.rlu.0000204200.66112.a9

[16] La Rosa FG , Flaig TW , Wilson S , et al. 2007. Sarcoidosis in a man with renal carcinoid tumor. Oncology (Williston Park) 21(13):1593–1596. 18179048

[17] Bae SY , Jeon K , Koh WJ , et al. 2010. Concurrent endobronchial carcinoid tumor and sarcoidosis. Intern. Med. 49(23):2609–2612. 21139301 10.2169/internalmedicine.49.4032

[18] Watanabe T , Sado T , Notsuda H , et al. 2011. Coexistence of sarcoidosis and thymic carcinoid. Kyobu Geka 64(13):1176–1179. 22242297

[19] Rafiei P , Souza F , and Vijayakumar V . 2015. Sarcoidosis mimicking metastatic carcinoid on indium‐111 pentetreotide scintigraphy. Radiol. Case Rep. 6(1):483. 27307891 10.2484/rcr.v6i1.483 PMC4901025

[20] Lera Álvarez R , Inchaurraga Alvarez I , Fernandez Fabrellas E , et al. 2012. Association of carcinoid tumor and sarcoidosis. Arch. Bronconeumol. 48(12):469–471. 22341301 10.1016/j.arbres.2011.12.006

[21] Bhowmik S , Ammannagari N , Grethlein S , et al. 2013. When Occam's razor fails: a case of concomitant carcinoid and sarcoidosis. Am. J. Med. 126(12):e7–e8. 10.1016/j.amjmed.2013.07.009 24083645

[22] Kanapilly . 2014. Sarcoidosis with pulmonary parenchymal involvement and co‐existent endobronchial carcinoid. Kuwait Med. J. 46(1):73–75.

[23] Mungo N , Smith AK , Swenson J , et al. 2017. Questioning the connection between sarcoidosis and gastrointestinal carcinoid tumors. Am. J. Gastroenterol. 112:S965.

[24] Lolli I , Stasi E , Fucilli F , et al. 2017. Sarcoidosis mimicking metastatic progression of pancreatic neuroendocrine tumor: a case report. Medicine (Baltimore). 96(26):e7273. 28658123 10.1097/MD.0000000000007273 PMC5500045

[25] AlSamman S , and Samavati L . 2018. Concomitant lung carcinoid tumor and sarcoidosis. Crit. Care Med. 46(1):507.

[26] Yao JC , Hassan M , Phan A , et al. 2008. One hundred years after "carcinoid": epidemiology of and prognostic factors for neuroendocrine tumors in 35,825 cases in the United States. J. Clin. Oncol. 26(18):3063–3072. 18565894 10.1200/JCO.2007.15.4377

[27] Almuhaideb A , Papathanasiou N , and Bomanji J . 2011. 18F‐FDG PET/CT imaging in oncology. Ann. Saudi Med. 31(1):3–13. 21245592 10.4103/0256-4947.75771 PMC3101722

[28] Fallahi B , Manafi‐Farid R , Eftekhari M , et al. 2019. Diagnostic efficiency of ^68^Ga‐DOTATATE PET/CT as compared to ^99m^Tc‐octreotide SPECT/CT and conventional morphologic modalities in neuroendocrine tumors. Asia Ocean J. Nucl. Med. Biol. 7(2):129–140. 31380452 10.22038/AOJNMB.2019.39392.1263 PMC6661311

